# Screening for Distress in Routine Oncological Care—A Survey in 520 Melanoma Patients

**DOI:** 10.1371/journal.pone.0066800

**Published:** 2013-07-05

**Authors:** Carmen Loquai, Vera Scheurich, Nils Syring, Irene Schmidtmann, Stephan Rietz, Andreas Werner, Stephan Grabbe, Manfred E. Beutel

**Affiliations:** 1 Department of Dermatology, University of Mainz, Mainz, Rhineland Palatinate, Germany; 2 Department of Psychosomatic Medicine and Psychotherapy, University of Mainz, Mainz, Rhineland Palatinate, Germany; 3 Institute of Medical Biostatistics, Epidemiology and Informatics, University of Mainz, Mainz, Rhineland Palatinate, Germany; 4 Tumor Centre Rhineland-Pfalz, Mainz, Mainz, Rhineland Palatinate, Germany; The University of Hong Kong, Hong Kong

## Abstract

**Introduction:**

Despite the increasing incidence of melanoma little is known about patients' emotional distress associated with this disease. Supplemented by the problem list (PL), the distress thermometer (DT) is a recommended screening instrument to measure psychosocial distress in cancer patients. Our objective was to explore the acceptance and the feasibility of the DT and PL as a concise screening tool in an ambulatory setting for routine care and to elucidate determinants of distress in melanoma patients with regard to sociodemographic and clinical variables.

**Methods:**

Consecutive melanoma outpatients were asked to complete the DT with the PL prior to their scheduled consultation. Demographic and clinical data were obtained from the patients' charts. Clinical data included melanoma stage, time since diagnosis, previous treatment, current treatment, and other cancer disease.

**Results:**

Out of 734 patients recruited into the study, 520 patients (71%) completed both the DT and the PL. Forty-seven percent met the ≥5 cut-off score for distress. Younger and employed patients reported higher distress than older and retired patients. A cut-off score of ≥5 was closely associated with self-reported emotional sources of distress, with practical problems, especially at work, family problems (dealing with the partner), and physical problems like pain, appearance, getting around, and nausea. Apart from higher distress under current systemic treatment, no associations were found between distress and clinical data.

**Conclusion:**

The DT together with the PL seems to be an economically reasonable screening tool to measure psychosocial distress in melanoma patients. In particular, younger melanoma patients who are currently employed are prone to experience distress at some point after diagnosis, but there appears to be almost no association between clinical data and the extent of distress. To characterize the impact of distress on disease outcome and quality of life in melanoma patients, further research is needed.

## Introduction

The National Comprehensive Cancer Network (NCCN) defines distress as a “multifactorial, unpleasant emotional experience of a psychological (cognitive, emotional), social, and/or spiritual nature that may interfere with the ability to cope effectively with cancer, its physical symptoms and its treatment” [1], [2]. Malignant melanoma is the sixth most frequent form of cancer in the USA, which has continuously increased reaching up to 22.2 per 100,000 population in 2008 [3] and usually appears during middle adulthood. Particularly common in fair-skinned populations [3], its occurrence and prognosis are strongly related to behavioral factors, especially sun exposure patterns and the use of indoor tanning booths. Despite a deepening understanding of melanoma tumor biology and promising advances in treatment, surprisingly little is known about the psychological impact melanoma has on patients' lives. While the vast majority of cases are detected at an early stage and therefore treated effectively, recurrence remains a significant risk over many years. Thus, patients suffering from malignant melanoma have to grapple with an ongoing threat. Regular aftercare is therefore recommended for 10 years under the current German melanoma S3 guideline [4], including psycho-oncological treatment.

Yet, distress in malignant melanoma has remained understudied. Estimates of distress vary widely in patients with malignant melanoma. In a systematic review [5], the proportion of participants scoring in the clinical range for anxiety based on the Hospital Anxiety and Depression Scale (HADS) ranged from 18 to 44%, for depression symptoms the range was 6 to 28%. Risk factors for heightened distress were female sex, younger age, the absence of a spouse or partner, and lower levels of education. Surprisingly, stage of disease was unrelated, but physical deterioration and visibility of body site were associated with altered body image and fear of distress [6]. Further associations of distress were found with lack of social support, negative cognitive appraisal and an avoidant coping style [7].

There is evidence that psychological distress is associated with decreased adherence to treatment regimes, lower quality of life, reduced enrollment in follow-up programs, delay in seeking medical advice, increased recurrence rates and mortality, and increased medical costs [8]–[18]. Psychological distress, however, is often overlooked by physicians for many reasons. Patients are often reluctant to ask for help because they fear being stigmatized for having a psychological problem. They do not want to distract physicians from curing their cancer by mentioning psychosocial needs or fear being seen as overly demanding or difficult [13], [19]. Symptoms associated with distress, anxiety, or depression like loss of appetite, fatigue or insomnia might be confounded by symptoms of malignancy or treatment side effects, and the medical staff are not always trained or skilled in perceiving and discussing emotional problems [20]–[22]. As the treatment of melanoma has increasingly been shifted to ambulatory care settings, physician consultations are shortened, thus limiting time to explore emotional well-being. Therefore, the development of screening strategies to improve the detection and management of psychological distress has become even more important.

Recommendations for melanoma surveillance in German skin cancer centers include screening, evaluation, and treatment of distress of melanoma patients [23]. Distress has been studied by validated standardized screening tools, such as the HADS or the Brief Symptom Inventory (BSI) [24]–[27]. Despite their relative brevity, however, these multi-item measures still require more time than is available in busy outpatient skin cancer centers. To improve and implement psycho-oncological care in routine melanoma care programs, the development of brief screening tools to detect psychological distress and the identification of risk factors for distress are urgent needs. Recognizing the need for economical means to screen rapidly for distress in cancer patients, Roth and colleagues developed the single-itemed “Distress Thermometer” (DT) [28]. In order to identify the potential problems that can induce the distress reported, a problem list (PL) is often added covering the five domains of practical, family, emotional and physical problems, and spiritual/religious concerns.

This study was undertaken to explore the acceptance and feasibility of the DT and PL as a brief screening tool in an ambulatory setting for routine care, as determined by the rate of completed questionnaires. We wanted to elucidate the prevalence of distress and problem areas in melanoma patients. We expected that heightened distress is associated with a higher load of problems. Based on previous studies, we hypothesize distress to be increased in younger, female and single living patients with a more recent diagnosis and under current treatment.

## Methods

### Participants

The study participants were consecutive melanoma patients attending the Skin Cancer Center of the University of Mainz Medical Center. The inclusion criteria were histologically proven diagnosis of melanoma, age of at least 18 years, the ability to read and understand the questionnaires, and the patient's consent to participate. Using a cross-sectional design, patients were recruited at all stages of disease and treatment during aftercare. Demographic and clinical data were obtained by linking the patient's questionnaire with information stored in the patient's chart. The demographic variables considered were age, gender, employment state, health insurance, and marital state. Clinical data included melanoma stage, time since diagnosis, stage of treatment (pretreatment, current treatment), multiple melanomas, and other cancers.

### Procedure

Patients were approached in the waiting areas at the center prior to a scheduled outpatient visit. After an explanation by trained nurses, participants were asked to complete the DT and PL.

### Measures

#### Distress Thermometer and Problem List

The DT is a single-itemed self-reported, pencil and paper measure consisting of a line with a 0–10 scale anchored at the 0 point indicating “no distress” and a scale point 10 indicating “extreme distress”. Patients are instructed to circle the number that best describes the level of distress during the past week. The DT is simple to score and easy to interpret, and since developed in 1998 it has been used and validated in numerous clinical studies and has been recommended as a screening module for distress by the NCCN Panel. In a mixed German sample of cancer patients undergoing rehabilitation, a cut-off score of 5 yielded the best discrimination for high levels of anxiety or depression (based on the HADS) with a sensitivity of 97% and a specificity of 41% [29]. Thus, the internationally recommended cut-off score of 5 was used in this study.

The PL was developed by the Distress Management Guidelines Panel of the NCCN. It consists of 35 problems commonly experienced by cancer patients in five categories (practical problems, family problems, emotional problems, spiritual-religious concerns, and physical problems). Patients indicate whether or not (“yes-no”) they have experienced any of those problems in the past 7 days.

### Statistical Analysis

The acceptance and feasibility of the DT and PL as an extremely concise screening tool in an ambulatory setting for routine care of melanoma was determined by the proportion of completed questionnaires. Descriptive statistics were used to characterize the sample with regard to demographic and clinical variables. Following the recommendations of Mehnert et al. [29], we considered a patient as highly distressed when DT ≥5.

To determine the association of DT with demographics and clinical characteristics, we fitted cumulative logit models assuming proportional odds for each variable. This type of model takes into account the ordinal scale of the DT and assumes that odds ratios for each predictor are constant over all possible dichotomizations [30]. It is a generalization of logistic regression to an ordinal outcome.

The association of the DT with PL items was described by fitting proportional odds cumulative logit models for each item, adjusting for demographic variables found to be associated with the DT. To assess the joint influence of PL items on the DT, a proportional odds cumulative logit model was fitted and variables were selected using backward selection. We considered models starting a) with all PL items, b) with the numbers of problems of specific types, and c) the overall number of problems; again we adjusted for demographic variables found to be associated with the DT. The analyses were performed using GraphPad Prism 5.0 and SAS 9.3.

### Ethics statement

Approval was received from the Ethic Committee of Rhineland-Pfalz, Germany.

## Results

### Patient characteristics

**Figure 1:**
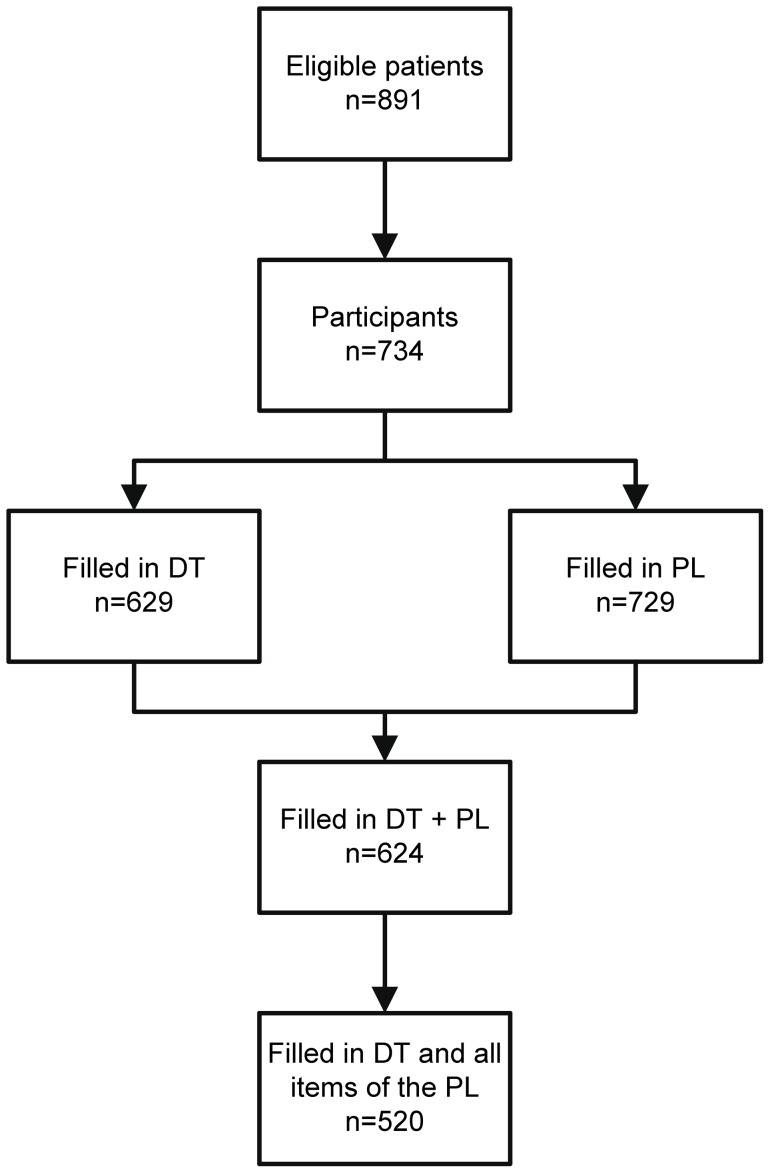
Participant flow, details of study recruitment.

Out of a total of 891 patients with melanoma visiting the skin cancer center during the study accrual period, 734 (82%) patients agreed to participate in the survey. Of these, 629 (86%) patients scored the DT and 729 (99%) patients filled in the PL. Both screening measures were filled in by 624 patients (85%). One or more (on average 3.18) items of the PL were omitted by 147 (20%) of the 729 patients who had filled in the PL. Both the DT and the PL were complete for 520 of 734 recruited patients (71%) (Figure 1). The statistical analysis has been restricted to those 520 patients who completed both the DT and all items of the PL.

The patient characteristics are summarized in Table 1 and Table 2. The average age of the patients included in the analysis was 58.5 (range 18–89, SD 14.0) years. Two hundred forty-three (47%) patients were female, and 277 (53%) were male. Patients with all stages of melanoma according to the classification of the American Joint Committee on Cancer (AJCC) 2009 were included: Melanoma in situ: 26 (5%), stage I/II: 401 (77%), stage III: 69 (13%) and stage IV: 24 (5%).

**Table 1:** Patient Demography (n = 520).

	Total	
	N	%
**Sex**		
Male	277	53
Female	243	47
**Employment status**		
Working	223	43
Retired	171	33
Other	76	15
Unknown	50	10
**Insurance status**		
Public	431	83
Private	89	17
**Status of relationship**		
Married	363	70
Widowed	25	5
Single (including divorced and separated)	82	16
Unknown	50	10

**Table 2:** Patient clinical variables (n = 520).

	Total	
	N	%
**AJCC stage**		
In situ	26	5
I/II	401	77
III	69	13
IV	24	5
**Time since diagnosis**		
0–12 months	143	28
>12–24 months	81	16
>24–36 months	50	10
>36–48 months	40	8
>48–60 months	44	8
>60 months	162	31
**SLNB**		
No	361	69
Yes	159	31
**Lymph node dissection**		
No	452	87
Yes	68	13
**Surgery for metastases (not skin, not lymph nodes)**		
No	510	98
Yes	10	2
**Radiotherapy**		
No	508	98
Yes	12	2
**Type of systemic therapy**		
None	373	72
Interferon	132	25
Other^1^	15	3
**Patient under systemic therapy**		
No	468	90
Yes	52	10
**Multiple melanomas**		
No	487	94
Yes	33	6
**Other nonmelanoma skin cancer**		
No	487	94
Yes	33	6
**Other noncutaneous malignancy**		
No	478	92
Yes	42	8

1 Vaccination, Immuntherapy+Vaccination, Chemoimmuntherapy, Chemoimmuntherapy+Vaccination, Chemoimmuntherapy+Targeted Therapy, Interferon+Interleukin-2.

### Association between distress score, categorical and continuous variables

**Figure 2:**
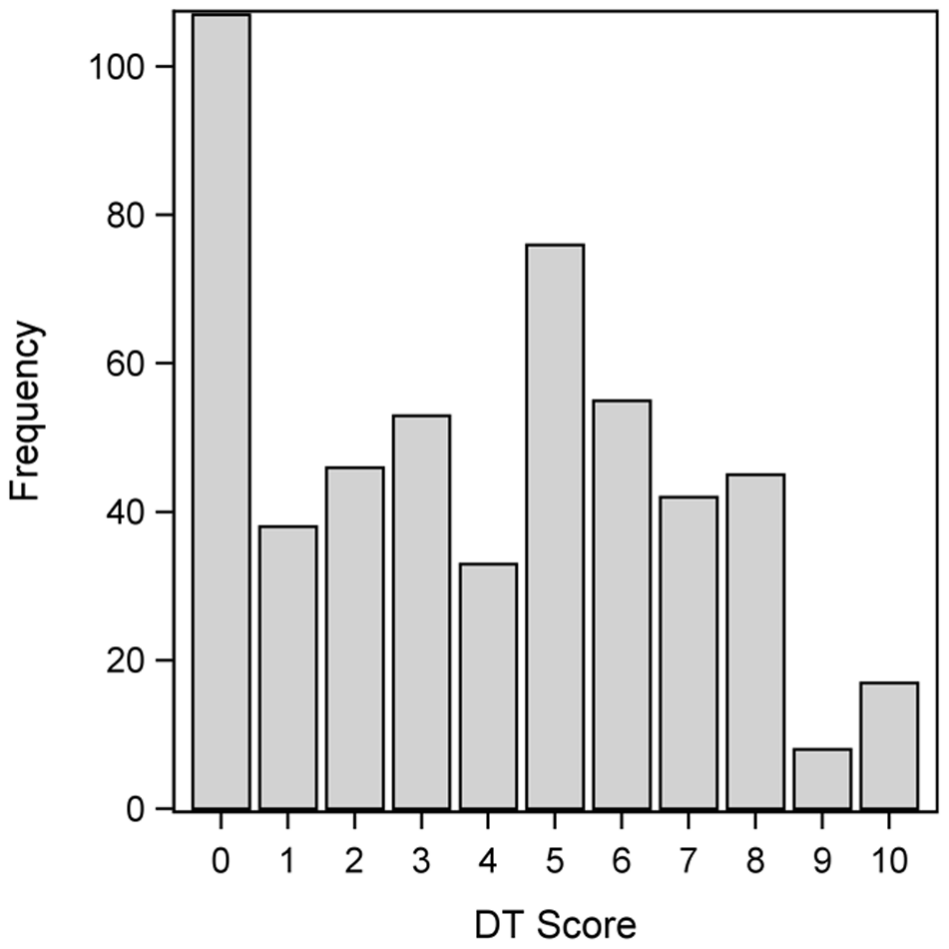
Frequency distribution of DT score among all patients completing the DT and the PL (n = 520).

The mean DT score was 3.9 (SD: 3.0), and the median score was 4, with a range from 0 to 10. Figure 2 summarizes the distribution of the DT scores. Two hundred seventy-seven (53%) patients reported a distress score of <5, whereas 243 (47%) scored ≥5 on the DT.

The association between patient characteristics and distress levels is shown in Table 3. Distress scores show a decreasing trend with age (OR = 0.97 per year, 95% CI [0.96; 0.98]). We found the DT to be associated with employment status, with retired patients having lower DT scores than working patients (OR = 0.51, 95% CI [0.36; 0.72]). Patients under systemic therapy had higher DT scores than patients not currently treated (OR = 1.91, 95% CI [1.16; 3.16]).

**Table 3:** Association of demographic and clinical variables with high distress (n = 520).

	DT score ≥5						
	No		Yes				
	N	%	N	%	Odds Ratio		95% confidence interval
**Sex**							
Male	153	55	124	45	1.0		
Female	124	51	119	49	1.34		[0.99; 1.81]
**Employment status**							
Working	100	45	123	55	1.0		
Retired	104	61	67	39	0.51		[0.36; 0.72]
Other	45	59	31	41	0.64		[0.41; 1.01]
Unknown	28	56	22	44	0.9		[0.52; 1.53]
**Insurance status**							
Public	225	52	206	48	1.0		
Private	52	58	37	42	0.79		[0.53; 1.17]
**Status of relationship**							
Married	192	53	171	47		1.0	
Widowed	14	56	11	44		0.75	[0.37; 1.54]
Single (including divorced and separated)	43	52	39	48		1.25	[0.82; 1.89]
Unknown	28	56	22	44		0.86	[0.51; 1.44]
**AJCC stage**							
In situ	13	50	13	50		1.07	[0.53; 2.13]
I/II	219	55	182	45		1.0	
III	35	51	34	49		1.21	[0.78; 1.89]
IV	10	42	14	58		1.41	[0.69; 2.89]
**SLNB**							
No	190	53	171	47		1.0	
Yes	87	55	72	45		0.99	[ 0.72; 1.37]
**Lymph node dissection**							
No	241	53	211	47		1.0	
Yes	36	53	32	47		1.14	[0.73; 1.78]
**Surgery for metastases (not skin, not lymph nodes)**							
No	272	53	238	47		1.0	
Yes	5	50	5	50		1.03	[0.35; 3.08]
**Radiotherapy**							
No	271	53	237	47		1.0	
Yes	6	50	6	50		1.53	[0.56; 4.15]
**Type of systemic therapy**							
None	196	53	177	47		1.0	
Interferon	72	55	60	45		1.1	[0.78; 1.55]
Other^1^	9	60	6	40		0.95	[0.38; 2.33]
**Patient under systemic therapy**							
No	254	54	214	46		1.0	
Yes	23	44	29	56		1.91	[1.16; 3.16]
**Multiple melanomas**							
No	257	53	230	47		1.0	
Yes	20	61	13	39		0.61	[0.33; 1.14]
**Other nonmelanoma skin cancer**							
No	259	53	228	47		1.0	
Yes	18	55	15	45		0.77	[0.41; 1.42]
**Other noncutaneous malignancy**							
No	254	53	224	47		1.0	
Yes	23	55	19	45		0.98	[0.56; 1.70]

1 Vaccination, Immuntherapy+Vaccination, Chemoimmuntherapy, Chemoimmuntherapy+Vaccination, Chemoimmuntherapy+Targeted Therapy, Interferon+Interleukin-2.

In order to assess the joint influence of demographics and clinical characteristics, we fitted a multivariate cumulative logit model and found that it was sufficient to include age; adding employment status or therapy status did not improve the model. Other sociodemographic variables such as sex, health insurance, or marital status did not affect distress levels. The detailed results are included in Table 3.

Interestingly, no further association with the distress score was found. In particular, no association exists between disease stage and distress level. Other melanoma-associated parameters such as type of treatment, presence of multiple melanomas, or another cancer diagnosis did not influence distress score, either. Shorter time since diagnosis was not associated with higher distress scores (OR = 1.0 per year, 95% CI [0.96; 1.03]).

### Distress score and Problem list

Table 4 provides an overview of the problems that were mentioned most frequently in the problem list, stratified by distress score (high versus low). The strength of association is measured by the odds ratio, adjusted for age.

**Table 4:** Association of problems indicated with high/low distress (n = 520).

	Problem present								Distress (high vs. low)^1^	
	No				Yes					
	DT score < 5		DT score ≥ 5		DT score < 5		DT score ≥ 5			
Problem	N	%	N	%	N	%	N	%	Odds Ratio	95% confidence interval
At least one problem	86	81	20	19	191	46	223	54	6.09	[4.03; 9.21]
**Practical problems**										
At least one practical problem	236	62	147	38	41	30	96	70	3.08	[2.14; 4.42]
Child care	273	55	224	45	4	17	19	83	2.60	[1.24; 5.46]
Housing	271	55	226	45	6	26	17	74	3.58	[1.71; 7.49]
Insurance/financial	264	55	217	45	13	33	26	67	2.54	[1.43; 4.53]
Mobility/transportation	264	55	219	45	13	35	24	65	1.73	[0.96; 3.10]
Work/school	252	58	181	42	25	29	62	71	3.25	[2.12; 4.98]
**Family problems**										
At least one family problem	259	60	175	40	18	21	68	79	4.53	[2.96; 6.94]
Dealing with children	269	55	223	45	8	29	20	71	2.15	[1.10; 4.19]
Dealing with partner	268	57	205	43	9	19	38	81	5.81	[3.36; 10.06]
Dealing with parents	269	56	215	44	8	22	28	78	2.63	[1.44; 4.80]
**Emotional problems**										
At least one emotional problem	180	72	71	28	97	36	172	64	4.51	[3.25; 6.25]
Depression	263	58	193	42	14	22	50	78	4.76	[2.96; 7.66]
Fear	246	62	153	38	31	26	90	74	4.33	[2.97; 6.33]
Nervousness	226	64	128	36	51	31	115	69	3.85	[2.74; 5.40]
Sadness	248	61	159	39	29	26	84	74	4.14	[2.82; 6.09]
Worries	235	65	129	35	42	27	114	73	4.19	[2.92; 6.00]
Loss of interest in daily activities	266	55	218	45	11	31	25	69	3.95	[2.16; 7.22]
**Physical problems**										
At least one physical problem	119	71	48	29	158	45	195	55	3.25	[2.32; 4.55]
Appearance	271	56	213	44	6	17	30	83	3.12	[1.70; 5.71]
Bathing/dressing	269	54	228	46	8	35	15	65	2.06	[0.99; 4.30]
Breathing	261	56	204	44	16	29	39	71	2.94	[1.79; 4.82]
Changes in urination	255	54	217	46	22	46	26	54	1.64	[0.97; 2.77]
Constipation	266	54	224	46	11	37	19	63	2.05	[1.07; 3.92]
Diarrhea	265	54	224	46	12	39	19	61	2.04	[1.08; 3.86]
Eating	253	54	219	46	24	50	24	50	1.96	[1.17; 3.33]
Fatigue	212	61	135	39	65	38	108	62	2.29	[1.65; 3.18]
Feeling swollen	248	55	205	45	29	43	38	57	1.90	[1.21; 2.99]
Fevers	273	53	240	47	4	57	3	43	0.70	[0.19; 2.61]
Getting around	260	56	207	44	17	32	36	68	3.47	[2.09; 5.76]
Indigestion	264	55	218	45	13	34	25	66	1.88	[1.05; 3.35]
Mouth sores	262	54	223	46	15	43	20	57	2.28	[1.25; 4.17]
Nausea	269	55	219	45	8	25	24	75	3.16	[1.67; 5.95]
Nose dry/congested	245	56	196	44	32	41	47	59	1.75	[1.15; 2.66]
Pain	247	60	164	40	30	28	79	72	3.31	[2.26; 4.85]
Sexual	255	55	209	45	22	39	34	61	1.85	[1.14; 3.01]
Sleep	221	59	153	41	56	38	90	62	2.02	[1.43; 2.83]
Tingling in hands/feet	234	57	180	43	43	41	63	59	1.70	[1.17; 2.47]
**Spiritual problems**	274	54	238	46	3	38	5	63	4.52	[1.32; 15.47]
**Other problems**	259	55	210	45	18	35	33	65	2.34	[1.41; 3.90]

1 The odds ratios and corresponding 95% confidence intervals in this table are obtained by fitting a proportional odds cumulative logit model for each problem list item, adjusting for age. The odds ratios given here describe the odds for high distress in the presence of a problem relative to the odds in the absence of the problem.

Most patients mentioned at least one physical problem (n = 353; 68%), and at least one emotional problem was reported by 269 (52%) patients. Practical problems were mentioned by 137 (26%), family problems were indicated by 86 (17%) patients, and other problems by 51 (10%) patients. Spiritual problems were rarely mentioned (n = 8; 1.5%).

Patients reporting more problems were more likely to score highly on the DT. This applied to emotional problems in general (OR = 4.51, 95% CI [3.25; 6.25]) and to each emotional problem in particular (nervousness (OR = 3.85, 95% CI [2.74; 5.40]), worries (OR = 4.19, 95% CI [2.92; 6.00]), fear (OR = 4.33, 95% CI [2.97; 6.33]), sadness (OR = 4.14, 95% CI [2.82; 6.09]), depression (OR = 4.76, 95% CI [2.96; 7.66]), and loss of interest in daily activities (OR = 3.95, 95% CI [2.16; 7.22]). This was also true for family problems in general (OR = 4.53, 95% CI [2.96; 6.94]) and for each family problem in particular (Table 4). The most pronounced association of a family problem with the DT was observed in “dealing with partner” (OR = 5.81, 95% CI [3.36; 10.06]). In practical problems, work/school problems and problems with child care were associated strongly with high distress scores. Problems with housing also showed a strong association with the DT, but were observed only in 23 patients (4.2%).

In general, physical problems were also associated with high DT scores (OR = 3.25, 95%CI [2.32; 4.55]); the most pronounced associations were observed in problems with pain (OR = 3.31, 95% CI [2.26; 4.85]), appearance (OR = 3.12, 95% CI [1.70; 5.71]), getting around (OR = 3.47, 95% CI [2.09; 5.76]) and nausea (OR = 3.16, 95% CI [1.67; 5.95]). Spiritual problems showed more than a random association with high DT scores (OR = 4.52, 95% CI [1.32; 15.47]; however, they were rare in our patients. Fevers, changes in urination and problems with bathing/dressing did not show any association with DT scores.

**Figure 3:**
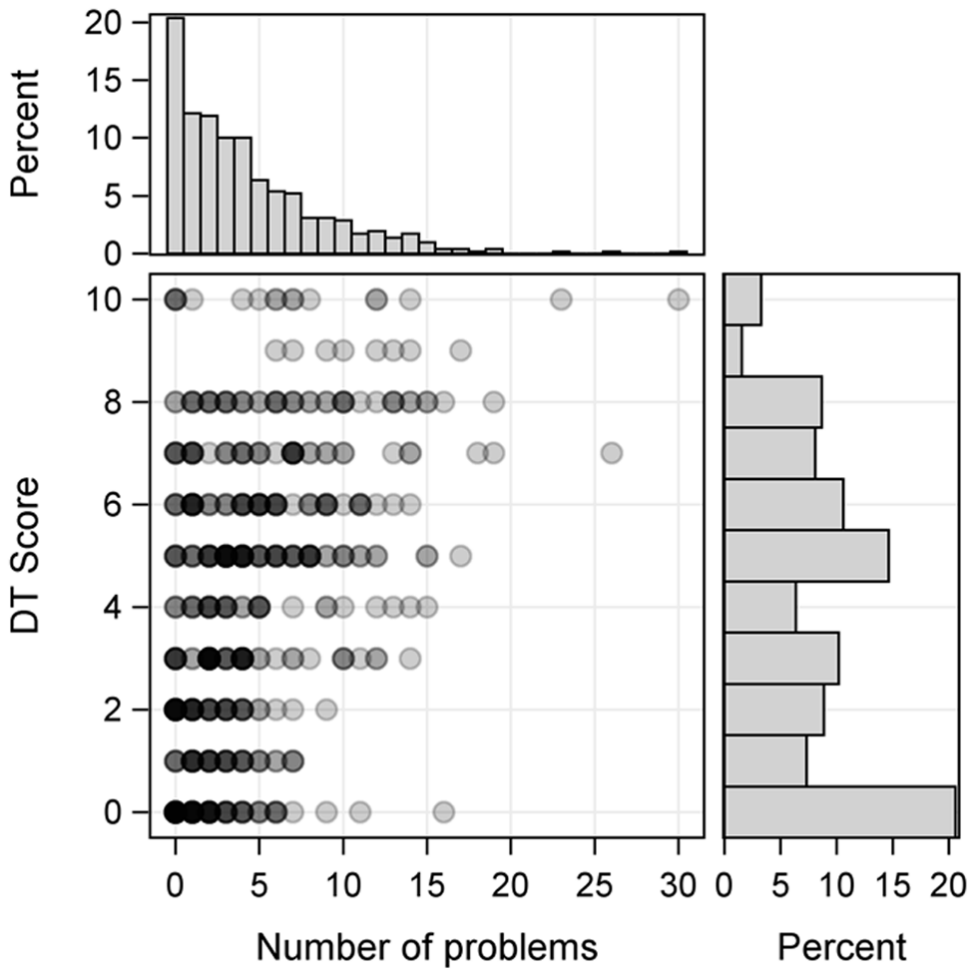
Joint distribution of DT scores and number of problems. The scatter plot (bottom left) depicts the association of the DT score and number of problems. The darkness of the dots indicates the number of patients having a particular combination of number of problems and the DT score. Dark shades correspond to many patients, light shades correspond to few patients. The histograms for the number of problems (top left) and for the DT score (bottom right) show the marginal distributions of these two traits.

As expected, distress scores were associated with the number of problems indicated by patients (OR = 1.24 per problem, 95%CI [1.19; 1.29]; i.e. patients with multiple problems had higher distress scores (Figure 3).

We modeled the joint influence of problems on the DT using a cumulative logit model, adjusting for age and applying backward selection. The first considered model started with all problem list items. Work/education related problems, problems dealing with partner, fear, nervousness, worries, breathing problems and pain remained in the selected model. When this model was fitted, we found 72% concordant pairs. The model selected when starting with the numbers of specific problems, i.e. practical, emotional, physical, spiritual, and family problems, contained the number of practical problems (OR = 1.35 per problem, 95% CI [1.10; 1.67], number of emotional problems (OR = 1.66 per problems; 95% CI [1.48; 1.87], and number of physical problems (OR = 1.95 per problem, 95% CI [1.37; 2.78]); here we found 71.7% concordant pairs. When only considering the overall number of problems, we found similarly good classification properties: 70.4% concordant pairs.

## Discussion

In general melanoma patients seem to be highly motivated to participate in psycho-oncological screening programs as 82% of the patients agreed to participate in this survey. Of the patients willing to participate, 71% filled in both screening measures, the DT and the PL, completely. Compared to the literature, which reports DT participation rates of about 80% to 90% [31], [32], our data are consistent. In the studies where DT with PL were combined, there are no clear data regarding the completeness of the PL, as often imputations were made for missing data [32].To our knowledge we have presented this kind of data for the first time in detail within a patient collective of 734 participating patients. We found a high motivation to participate in general as well as to fill in both the DT and the PL screening measures. However, there were some problems in filling in the complete survey with missing data in 29% of participating patients (average 3.18 missing items per patient). We have no information on why those patients skipped some items as we could not find any pattern of missing single items. Potential reasons could be that patients found some items not applicable for themselves or not important enough to mention.

The mean distress score in our melanoma patients was found to be 3.9, with 47% of patients reporting distress intensity between 5 and 10 on the DT. The mean score and percentages fell in the middle range reported in previous studies with ambulatory care cancer patients (mean of DT score of previous studies: 2.47–4.7) [31], [33]–[35].

In our sample, elevated distress scores were not associated with most disease-specific melanoma aspects such as previous treatment, localization of the tumor, tumor stage, or time since diagnosis. Only current treatment was associated with an increased distress, which is supported by the literature; a considerable number of our patients received adjuvant interferon alpha treatment, a treatment known to affect several aspects of quality of life [36], [37]. Apart from younger age and employment state, there was no association of distress with demographic data, including gender, health insurance, or marital state. This finding could indicate that melanoma patients are not strongly distressed due to their disease. Indeed, compared to other cancer entities, long-time melanoma survivors as well as prostate cancer patients seem to perform better [27]. To assess the impact of melanoma-specific distress, studies including distress data from the general population should be initiated.

Melanoma patients differ from other cancer patients in several aspects. On average, melanoma patients are younger than other cancer patients. This implies that diagnosis usually occurs at a time when most patients are still active at work, have to care for their children or pay off their mortgage. Being confronted with a possibly life threatening disease in a period of life characterized by career and family duties may cause existential concerns, particularly in younger patients [1], [38].

Most melanoma patients are diagnosed at an early stage of the disease without the need for further adjuvant treatment after initial surgery. The absence of any signs of disease together with a lack of physical impairment may distract the patient from the cancer diagnosis and may enable the patient to cope well with his or her disease. Indeed, most patients with melanoma seem to cope well; however even patients with early-stage disease have to deal with the possibility of recurrence or systemic spread, which is highest in the first 3 years but can also occur more than 10 years after diagnosis. This underlying fear could explain the distress of our patients regardless of disease stage, suggesting that the possibility of disease recurrence/metastasis is the major stress factor.

Accordingly, the most prevalent problems of our patients who had distress scores indicating psychosocial referral were of emotional nature. Consistent with published data [39], in addition to other emotional problems worries and fear were strongly associated with high distress. In a recent survey of 1490 cancer patients, Mergenthaler et al. [31] found that 97% of patients appreciated speaking with their doctor about their distress, and 56% felt better than usual after this consultation. As distress in our patients was not only associated with emotional problems but also with practical, family, and physical problems like dealing with their partner, problems at work, nausea, or pain, the role of the primary physician to meet and treat unmet needs should not be underestimated. The DT with the PL can help identify distress sources and stimulate doctor-patient communication. The primary physician than can act as a gate keeper, who refers the patient to the specific professional he or she needs: for physical problems to physicians or nurses, for emotional problem to psycho-oncologists, and for practical problems to social workers.

There are certain limitations in our study that should be considered. First, it has a cross-sectional design. A longitudinal study could probably better address patients' needs during their disease process and identify patients in need for professional psychosocial support. Second, DT and PL have only been studied in cancer patients but not in the general population, and third, we did not investigate the influence of comorbidities, especially chronic diseases other than cancer and mental illnesses. Therefore the impact of melanoma disease on distress still has to be defined in further studies considering these issues.

## Conclusions

The present findings have important implications for future research and clinical practice. First, the results suggest that the DT and PL may be used to identify distressed melanoma patients. It seems to be an economically reasonable initial measure and helps to better identify patients who would actually profit from further psychosocial intervention. Even though melanoma patients regardless of stage seem to cope well with their disease, younger patients who are currently employed and patients under current systemic treatment should be followed more cautiously. As distress can be influenced not only by disease-specific items but also by problems of daily living, comorbidities, a patient's history, or socioeconomic issues, a patients' concerns depicted by the DT and PL should stimulate doctor-patient communication and help to guide the patient to psychosocial professionals according to the patient's needs.
